# Enhancing Radiology Request Form Completeness With a Standardized Tool and Targeted Education: A Two-Cycle Clinical Audit

**DOI:** 10.7759/cureus.99549

**Published:** 2025-12-18

**Authors:** Mohaira Ishag Yagoob Shaddad, Manhal Eisa Galal Eisa, Esra Abdalla, Ahmed Nabil Mahmoud Mohamed, Shimaa T Elshikh, Ayat Alfaki, Ahmed Mohamedosman Abdalla Mohamed, Zainab Mohamed, Einas Mohammed Ibrahim Mohammed, Sulafa A. H Baleela, Mohamed Mohamed-Sharif, Lina Ahmed, Ahmed Hisham Mohammedosman Kheiralla, Badawi ElAmin Ahmed ElKhalifa, Riem Eltahir, Mazin Elyas Alawad Mohammed, Mohamed Saifeldin Sharif Hussein, Mohamed Almustafa Magdi M Mohamed Ahmed, Tasneem Sulieman M Tahameed, Faris Jamalaldeen Mohammed Hamed

**Affiliations:** 1 Emergency Department, Bashair University Hospital, Khartoum, SDN; 2 General Surgery, Al-Manaqil Teaching Hospital, Manaqil, SDN; 3 Internal Medicine, Cork University Hospital, Cork, IRL; 4 Critical Care Medicine, SEHA Salma Rehabilitation Hospital, Abu Dhabi, ARE; 5 Resident Physician, University of Bahri, Khartoum, SDN; 6 College of Medicine and Health Science, Omdurman Islamic University, Omdurman, SDN; 7 Internal Medicine, Dongola Specialized Hospital, Dongola, SDN; 8 Trauma, Gezira University, Wad Madani, SDN; 9 General Practice, Heartlands Hospital, Birmingham, GBR; 10 Emergency Department, Al-Manaqil Teaching Hospital, Manaqil, SDN; 11 Surgery, Al-Manaqil Teaching Hospital, Manaqil, SDN; 12 Neonatology, Heartlands Hospital, Birmingham, GBR; 13 Emergency Department, Atbara Teaching Hospital, Atbara, SDN; 14 Surgery, Al-Razi University, Cairo, EGY; 15 Otolaryngology, Sudan Medical Specialization Board, Khartoum, SDN; 16 General Practice, Al-Neelain University, Khartoum, SDN

**Keywords:** clinical audit, diagnostic safety, documentation quality, multidisciplinary communication, radiology request forms, royal college of radiologists, sudan

## Abstract

Background: Incomplete radiology request forms (RRFs) hinder diagnostic accuracy, delay patient management, and increase the risk of unnecessary imaging. This issue is particularly relevant in resource-limited healthcare settings where RRFs represent the primary communication tool between clinicians and radiology departments.

Objectives: To evaluate the completeness of RRF documentation at Bashair University Hospital and assess the impact of implementing a standardized form supported by targeted clinician education.

Methods: A prospective closed-loop clinical audit was conducted over two cycles, with 50 RRFs reviewed in each phase. Cycle 1 was performed over two weeks in May 2025, followed by a one-month intervention that introduced a structured Royal College of Radiologists (RCR)-compliant request form and delivered educational sessions. Cycle 2 was conducted over a subsequent three-month period. Data were extracted using a predefined checklist and analyzed using descriptive statistics and chi-square testing.

Results: Baseline documentation was markedly deficient across several critical parameters, including patient address 0 (0.0%), urgency 0 (0.0%), clinical background 8 (16.0%), and the stated clinical question 3 (6.0%). Following the intervention, statistically significant improvements were observed in address documentation 47 (94.0%), p < 0.0001; urgency 47 (94.0%), p < 0.0001; clinical background 42 (84.0%), p < 0.0001; and diagnostic question 41 (82.0%), p < 0.0001. Recording the referring clinician’s name also improved substantially from 9 (18.0%) to 47 (94.0%) (p < 0.0001). The patient's name was consistently documented at 50 (100.0%) in both cycles. Conversely, telephone contact information remained poorly documented (1, 2.0%, p = 1.000), and documentation of the requested imaging modality showed no statistically significant improvement (p = 0.5579).

Conclusion: The implementation of a standardized RRF and focused educational intervention substantially improved documentation quality and compliance with RCR standards. These findings demonstrate the feasibility and effectiveness of low-cost interventions in optimizing communication, enhancing diagnostic safety, and reducing unnecessary imaging. Continued monitoring, reinforcement of contact information fields, and periodic re-audits are recommended to sustain improvements.

## Introduction

According to the National Institute for Health and Care Excellence (NICE), a clinical audit is a systematic process that evaluates current practice against defined standards and implements targeted improvements to enhance patient care [1]. In the United Kingdom, clinical audits have become an integral driver of healthcare quality and safety, with repeated audit cycles shown to strengthen performance and promote sustained improvement over time [2].

Radiology request forms (RRFs) are critical communication tools that link referring clinicians with radiology staff. Incomplete or inaccurate documentation, such as missing clinical history, unclear imaging rationale, or incorrect patient identifiers, can compromise diagnostic accuracy, delay investigations, and expose patients to unnecessary or inappropriate imaging. Handwritten forms often suffer from poor legibility, further increasing the risk of misinterpretation and workflow disruption [3].

The Royal College of Radiologists (RCR) emphasizes that RRFs must be precise, legible, and sufficiently detailed to support appropriate imaging selection and accurate interpretation [4]. Adequate clinical context and a clear diagnostic question help radiologists assess the justification of an examination and optimize imaging strategy [5,6]. Previous studies indicate that up to one-fifth of imaging requests may be clinically unwarranted due to poor documentation or insufficient justification, underscoring the medicolegal significance of well-completed RRFs [7].

In many African settings, persistent gaps in radiology training and shortages in the radiology workforce heighten the need for high-quality referral documentation [8]. Despite international recommendations, RCR standards have not been consistently applied within Sudan. To date, no audit has evaluated the quality of radiology request form documentation at Bashair University Hospital.

The objective of this audit was to assess the completeness of radiology request form documentation at Bashair University Hospital and to improve documentation quality through the introduction of a standardized request form alongside targeted educational interventions.

## Materials and methods

This prospective, closed-loop clinical audit was conducted in the Radiology Department at Bashair University Hospital in Sudan, a tertiary healthcare institution serving a wide catchment population. The overarching aim was to assess the completeness, accuracy, and clinical appropriateness of radiology request forms (RRFs) submitted by referring clinicians and to evaluate the impact of an intervention aimed at improving adherence to documentation standards recommended by the Royal College of Radiologists (RCR). The audit followed a structured methodology consisting of baseline assessment, intervention implementation, and re-audit.

Pre-intervention assessment (cycle 1)

The initial audit cycle was conducted over a consecutive two-week period in May 2024. Fifty RRFs were sampled using a systematic random sampling approach in which every third form submitted to the radiology department was selected. No inclusion restrictions were applied in terms of patient age, specialty, or urgency, allowing assessment of a representative cross-section of clinical practice. Each form was examined for the presence and clarity of essential documentation parameters, including patient identifiers, clinical history, referring location, imaging modality requested, urgency, and referring clinician details. Several systemic deficiencies were noted during this phase, including incomplete demographic data, missing diagnostic queries, and vague clinical summaries, all of which had potential implications for radiological interpretation quality.

Root cause analysis

Following identification of baseline deficiencies, a structured root cause analysis was conducted using informal staff interviews, observational notes taken during routine radiology workflow, and review of departmental documentation pathways. Several contributory factors were identified. First, the existing request form lacked defined fields, prompting clinicians to omit information or write brief, nonspecific notes. Second, there was an absence of standardized documentation practices between departments, leading to variability in formatting and prioritization of information. Third, clinicians demonstrated an inconsistent understanding of the information radiologists require to produce high-quality reports. Additionally, the audit team observed that insufficient clinical detail frequently resulted in ambiguous radiological conclusions, prompting repeat imaging and unnecessary resource expenditure. These findings underscored the need for structured educational reinforcement and standardized document formatting.

Intervention strategy

A multi-modal intervention was designed and implemented over the following one-month period. A new structured RRF was developed by the audit team in alignment with RCR guidelines, incorporating clearly labelled sections for clinical history, diagnostic question, modality justification, patient location, urgency category, and clinician contact details. The completeness checklist used to evaluate each form was developed from RCR minimum documentation standards and consisted of 14 mandatory items. The redesigned form was printed, approved by hospital administration, and distributed across emergency, inpatient, and outpatient departments to ensure consistent use.

In parallel, an educational program was delivered to clinicians from multiple specialties. The sessions consisted of a 15-minute orientation covering RCR documentation standards, common errors identified during Cycle 1, radiation justification principles, and practical demonstrations of completing the new form. Teaching occurred through PowerPoint-assisted departmental meetings, focused small-group discussions, and one-to-one demonstrations. Instructional posters reinforcing essential documentation steps were displayed in clinical areas. Staff were encouraged to document explicit clinical questions rather than broad symptom descriptions.

Post-intervention reassessment (cycle 2)

Following the intervention, the re-audit cycle was conducted consecutively over a three-month period to allow sufficient exposure and adoption of the redesigned form. Fifty newly submitted RRFs were sampled using the same systematic random sampling method as in Cycle 1 and evaluated according to identical criteria. The extended assessment period enabled evaluation of early uptake and short-term sustainability of improvements.

Data extraction

All completed forms were physically retrieved from the radiology archive, and relevant information fields were manually transcribed by the audit team into a data collection spreadsheet specifically designed for this project. Each field was scored dichotomously (present/absent), with partial entries (e.g., illegible names or incomplete clinical histories) classified as absent to reinforce documentation quality. Audit parameters were selected based on RCR minimum requirements for imaging justification, clinical safety, and accurate patient identification.

Data analysis

Data analysis was performed using Microsoft Excel® 2016 (Microsoft Corporation, USA). Each field was assigned a binary numerical value (1 = present, 0 = absent) to allow quantitative comparison. Frequencies and proportional percentages were calculated for each parameter across both cycles. Improvements were expressed as percentage point differences between baseline and re-audit values. Graphical representations, including clustered bar charts and pie charts, were generated to visually demonstrate improvement trends and highlight persistent areas of deficiency.

No inferential statistical testing was performed due to the descriptive nature of the dataset, categorical structure of variables, and sample size constraints; however, descriptive analysis was deemed sufficient to determine clinically meaningful change. Cycle 2 data were further stratified by modality type (X-ray, ultrasound, echocardiography, computed tomography) to assess whether the introduction of CT services influenced documentation completeness. Comparative performance charts were subsequently included in the results to facilitate transparent interpretation.

Evaluation process and quality control

To enhance inter-rater reliability, four authors independently evaluated all RRFs. Where discrepancies occurred, such as disagreements on legibility or classification, adjudication was performed by a senior reviewer unaffiliated with the initial scoring process. Consensus decisions were then documented and incorporated into the final dataset, thereby minimizing subjective bias.

Ethical considerations

Ethical approval for this audit was granted by the Institutional Ethics Committee at Bashair University Hospital (IRB number: BTH-1742). No patient contact occurred at any stage, and all personal identifiers were removed prior to analysis. The audit adhered to institutional confidentiality policies and complied with ethical standards governing retrospective documentation review.

## Results

A total of 100 radiology request forms were reviewed, with 50 forms analyzed in each audit cycle. The baseline assessment demonstrated poor compliance with several essential documentation parameters. For example, 0 (0.0%) of Cycle 1 forms recorded a patient address, compared to 47 (94.0%) in Cycle 2, representing a statistically significant improvement (χ² = 83.269, p < 0.0001). Documentation of patient age increased markedly from 2 (4.0%) to 49 (98.0%) (χ² = 83.012, p < 0.0001), while the recording of patient location improved from 4 (8.0%) to 48 (96.0%) (χ² = 72.621, p < 0.0001).

Clinically relevant fields demonstrated similar gains. The inclusion of clinical background information rose from 8 (16.0%) in Cycle 1 to 42 (84.0%) in Cycle 2 (χ² = 42.706, p < 0.0001), and documentation of the clinical question to be answered increased from 3 (6.0%) to 41 (82.0%) (χ² = 54.440, p < 0.0001). Priority (urgency) status showed a substantial improvement from 0 (0.0%) to 47 (94.0%) (χ² = 83.269, p < 0.0001), indicating better triaging and radiology workflow management.

Fields relating to the referring clinician also improved considerably. Documentation of the doctor’s name increased from 9 (18.0%) to 47 (94.0%) (χ² = 54.440, p < 0.0001), while the clinician’s signature rose from 21 (42.0%) to 49 (98.0%) (χ² = 33.784, p < 0.0001). The inclusion of the date improved from 20 (40.0%) to 47 (94.0%) (χ² = 29.824, p < 0.0001), supporting traceability and medicolegal robustness.

Two parameters did not show a statistically significant change. Telephone contact information increased only slightly from 0 (0.0%) to 1 (2.0%) (p = 1.000), remaining critically under-documented. Imaging modality decreased marginally from 50 (100.0%) to 48 (96.0%), a non-significant change (p = 0.5579), reflecting pre-existing high compliance and automated modality selection on redesigned forms. Patient name was consistently documented in both cycles at 50 (100.0%), yielding no observable difference (p = 1.000).

Overall, the second audit cycle demonstrated statistically significant improvement across nearly all parameters measured, strongly supporting the effectiveness of the standardized form and educational intervention in enhancing radiology request completeness (Table 1).

**Table 1:** Comparison of complete documentation in radiology request forms before and after intervention at Bashair University Hospital (n = 50 per cycle). This table summarizes the frequency and percentage of completed documentation fields across two audit cycles following the implementation of a standardized radiology request form and targeted educational intervention. Frequencies are presented first, followed by percentages in parentheses. Chi-square testing with continuity correction was used to assess the statistical significance of change between cycles. A p-value <0.05 was considered statistically significant.

Parameter	Cycle 1 (n=50)	Cycle 2 (n=50)	χ² value	p-value
Patient name	50 (100.0%)	50 (100.0%)	0.000	1.0000
Address	0 (0.0%)	47 (94.0%)	83.269	<0.0001
Age	2 (4.0%)	49 (98.0%)	83.012	<0.0001
Location of patient	4 (8.0%)	48 (96.0%)	72.621	<0.0001
Clinical background	8 (16.0%)	42 (84.0%)	42.706	<0.0001
Question to be answered	3 (6.0%)	41 (82.0%)	54.440	<0.0001
Priority (urgency)	0 (0.0%)	47 (94.0%)	83.269	<0.0001
Date	20 (40.0%)	47 (94.0%)	29.824	<0.0001
Doctor name	9 (18.0%)	47 (94.0%)	54.440	<0.0001
Doctor signature	21 (42.0%)	49 (98.0%)	33.784	<0.0001
Telephone	0 (0.0%)	1 (2.0%)	0.000	1.0000
Imaging modality	50 (100.0%)	48 (96.0%)	0.343	0.5579

## Discussion

Incomplete radiology request form (RRF) completion has been extensively established as a recurring worldwide issue impacting patient safety, workflow efficiency, and diagnostic accuracy [9]. Multidisciplinary cooperation is essential for the best patient care, and radiologists can choose the best imaging modalities and produce more insightful reports when they get comprehensive, context-rich referrals [4,7,9]. The RRF remains the main means of contact between radiologists and referring doctors in many healthcare settings, especially those with large patient volumes or low staffing [10]. Incomplete forms frequently cause reporting delays or result in less-than-ideal investigations, even when direct verbal or telephone communication may happen in certain situations.

The audit's conclusions showed significant shortcomings in the first evaluation cycle. Address, urgency, referring clinician information, and clinical questions were among the many crucial components that were missing. This pattern is in line with earlier research by Depasquale and Crockford [9] and Akinola et al. [11], which found that just 4% of fully completed RRFs met acceptable standards. However, after the intervention in our second cycle, 47 (94.0%) of the forms had a patient address, which was significantly greater than the 2.1% recorded by Akinola et al. [11] and higher than the 77% reported by Depasquale and Crockford [9]. Particularly for inpatients, where ward identification speeds up reporting return pathways and reduces transport delays, this improvement demonstrates the significance of structured fields in lowering omissions and facilitating more effective communication.

Throughout both cycles, the patient's name was continuously documented in 50 (100.0%) of the forms, demonstrating the continued need for accurate patient identification in safe medical practice. This result is consistent with that of Agi et al. [12], whose Nigerian study likewise reported universal compliance in patient name documentation. In contrast to the 100% reported by Agi et al. [12], only 3 (6.0%) of the forms in Cycle 1 included a clear clinical concern that needed radiological explanation. In addition to potentially resulting in improper imaging and lengthening patient wait times, the lack of clinical questions compromises diagnostic reasoning. This value increased to 41 (82.0%) after the intervention, demonstrating how well education and standardized forms work to increase referral clarity.

For radiologists to evaluate justification, customize imaging methods, and reduce needless radiation exposure, the clinical data supplied by referring clinicians is crucial [13]. Referring physicians are in charge of gathering previous diagnostic data, making sure that the right rationale is provided, and avoiding unnecessary radiation exposure, according to the European Radiation Protection Regulations [14]. Stronger adherence to these safety guidelines is seen by the rise in clinical background documentation from 8 (16.0%) to 42 (84.0%), which enhances diagnostic confidence and lowers the need for repeat exams.

There was still a severe lack of documentation regarding the contact details of recommending physicians. Only one form (2.0%) in all cycles included a phone number, which made it impossible to promptly clarify unexpected or inadvertent findings. The Royal College of Radiologists (RCR) guidelines, which require contact information to enable emergency communication, are directly at odds with this [4]. Persistent resistance to completing this section indicates the need for ongoing advocacy and strengthened accountability, even with greater process consistency.

Strengths

This audit demonstrated several important strengths. It used a structured, closed-loop, two-cycle methodology that allowed objective measurement of change following a well-defined intervention. The combination of a standardized RCR-aligned request form and focused clinician teaching represented a simple, low-cost, and highly scalable strategy, making the findings relevant to other low-resource environments. The audit also utilized clearly defined, measurable data fields, enhancing the precision and reproducibility of comparisons between cycles.

Limitations

Despite these strengths, several limitations should be acknowledged. This audit was conducted in a single center with a relatively small sample size of 50 forms per cycle, limiting broader generalizability. Although systematic random sampling was used, variations in clinician staffing or clinical workload may still have influenced documentation patterns. The possibility of a Hawthorne effect, where documentation temporarily improves due to awareness of being monitored, cannot be excluded. Additionally, although interventions improved multiple domains, some fields (particularly clinician phone numbers) remained persistently underreported, highlighting ongoing behavioral and system-level barriers. Finally, while the educational intervention was described, additional procedural details would further enhance external reproducibility.

Notwithstanding the general advancements, several restrictions must be noted. The fact that this audit was only carried out in one institution may limit its generalizability. Although it may not capture wider documentation heterogeneity, the sample size of 50 forms per cycle offers directional insight. Furthermore, temporary improvements in documentation behavior may have resulted from staff awareness of continuous monitoring (Hawthorne effect). Before extrapolating results to other facilities with varied resource capacity, imaging availability, or departmental structures, care should be taken because these data represent the operating environment, staffing patterns, and workflow of Bashair University Hospital.

The feasibility of improving documentation quality by standardization and teaching is nevertheless demonstrated by the notable gains seen across the majority of metrics after a very inexpensive intervention. Sustained improvement will depend on periodic re-auditing, continuous staff engagement, reinforcement of medicolegal responsibilities, and targeted efforts to improve consistently neglected fields such as clinician contact information.

## Conclusions

This closed-loop clinical audit showed that introducing a standardized, RCR-compliant radiology request form alongside targeted clinician education significantly improved the completeness of referral documentation at Bashair University Hospital. Marked gains were observed across key parameters, particularly clinical background, diagnostic question, urgency, and patient address, demonstrating the effectiveness of simple, low-cost interventions in strengthening documentation quality. Although the audit did not assess long-term sustainability or clinical outcomes, the consistent improvements across cycles support the value of structured forms and focused teaching in enhancing referral clarity. Continued monitoring, reinforcement of clinician contact details, and periodic re-audits are recommended. This approach provides a practical, reproducible model for improving RRF documentation in comparable resource-limited settings.
